# An *NFKB1* Promoter Insertion/Deletion Polymorphism Influences Risk and Outcome in Acute Respiratory Distress Syndrome among Caucasians

**DOI:** 10.1371/journal.pone.0019469

**Published:** 2011-05-09

**Authors:** Ednan K. Bajwa, Paul C. Cremer, Michelle N. Gong, Rihong Zhai, Li Su, B. Taylor Thompson, David C. Christiani

**Affiliations:** 1 Pulmonary and Critical Care Unit, Department of Medicine, Massachusetts General Hospital, Harvard Medical School, Boston, Massachusetts, United States of America; 2 Division of Critical Care Medicine, Department of Medicine, Montefiore Medical Center, Department of Epidemiology and Population Health, Albert Einstein College of Medicine, Bronx, New York, United States of America; 3 Department of Environmental Health, Harvard School of Public Health, Boston, Massachusetts, United States of America; Ohio State University Medical Center, United States of America

## Abstract

**Background:**

Nuclear factor-κB (NF-κB) is required for transcription of many pro-inflammatory genes and has been implicated in the pathogenesis of acute respiratory distress syndrome (ARDS). We hypothesized that a known functional polymorphism in the promoter of the *NFKB1* gene may affect susceptibility to and outcome from ARDS.

**Methods:**

A case control study was conducted among a cohort of patients admitted to the intensive care unit (ICU) with risk factors for the development of ARDS. 379 patients with ARDS and 793 at-risk controls were studied. Patients were followed for 60 days with development of ARDS as a primary outcome; ARDS-related mortality and organ dysfunction were secondary outcomes.

**Results:**

Patients homozygous for the 4 base pair deletion in the promoter of NFKB1 (*del/del*) did not have an increased odds ratio (OR) of developing ARDS in unadjusted analysis but were more likely to develop ARDS in the presence of a significant interaction between the *del/del* genotype and age (OR 5.21, 95% CI 1.35–20.0). In multivariate analysis, patients with ARDS and the *del/del* genotype also had increased 60 day mortality (HR 1.54, 95% CI 1.01–2.36) and more severe daily organ dysfunction (P<.001) when compared to ARDS patients with other genotypes.

**Conclusion:**

The *del/del* genotype is associated with an age-dependent increase in odds of developing ARDS. Patients with the *del/del* genotype and ARDS also have increased hazard of 60 day mortality and more organ failure.

## Introduction

The acute respiratory distress syndrome (ARDS) is a major cause of morbidity and mortality [1]. Common causes of ARDS include sepsis, pneumonia, aspiration, trauma, and multiple transfusions [2]. However, most individuals with these exposures do not develop ARDS [3]. Genetic variations in pro- and anti-inflammatory cytokines could account for some of this variability in risk, and several candidate genes involved in inflammation have been implicated in the development, severity, and mortality of ARDS [4]–[12].

Given its known functions in transcriptional regulation of chemokines and cytokines, Nuclear Factor Kappa-Beta (NF-κB) has been implicated in the pathogenesis of asthma, systemic inflammatory response syndrome (SIRS), and ARDS [13]. The NF-κB pathway is important for lung inflammation in mouse epithelial cells, and individuals with ARDS have increased activation of NF-κB in alveolar macrophages when compared to controls [14], [15].

Prior to stimulation, NF-κB dimers reside primarily in the cytoplasm as an inactive complex with nuclear factor-κ B inhibitor (IκB). In response to extracellular stimuli, IκB is phosphorylated. This phosphorylation allows the NF-κB dimer to dissociate and translocate to the nucleus where it can bind DNA promoter sequences of pro-inflammatory genes [16].

However, genes in the NF-κB family do not ubiquitously upregulate innate immunity. One example is *NFKB1*, a gene on chromosome 4q that encodes a 105 kD protein. This protein can undergo cotranslational processing to produce a 50 kd protein (p50). p50 contains the N-terminal Rel homology domain common to all NF-κB family members but lacks the COOH-terminal transactivation domain. The N-terminal Rel homology domain is responsible for dimerization and DNA binding while the COOH-terminal transactivation domain is necessary for positive regulation of gene expression. Since p50 lacks this COOH-terminal transactivation domain, homodimers may repress transcription [16]–[18].

A four base pair insertion/deletion polymorphism in the promoter of *NFKB1* (-94ins/delATTG, rs28362491) has been previously identified, and *in vitro* assays have demonstrated decreased promoter activity with the deletion allele [19]. Therefore, patients with the deletion allele may have decreased transcription of repressive p50 subunits and inappropriate upregulation of pro-inflammatory genes. Adamzik and colleagues demonstrated that this deletion allele was associated with worse lung injury scores but not increased mortality in patients with ARDS [10]. However, the study had low statistical power because of small sample size. Their study also did not assess whether the deletion allele conferred a risk for the development of ARDS because only patients with established ARDS were included.

Given the possible role of the NF-κB pathway in the inflammatory process of ARDS and the potential for the deletion polymorphism in the promoter of *NFKB1* to affect transcriptional regulation, we examined whether the insertion/deletion polymorphism in the promoter of *NFKB1* might contribute to susceptibility to ARDS. In addition, we examined ARDS-related mortality and organ dysfunction as secondary outcomes.

## Methods

### Study Enrollment and Design

**Figure 1:**
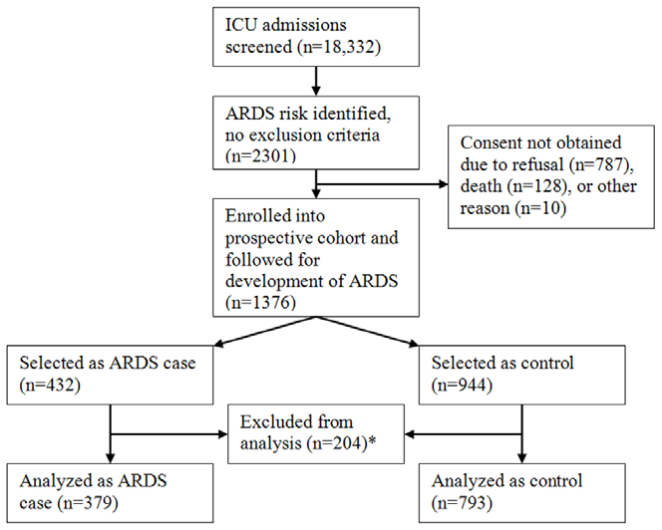
Schematic of patient selection and study design. *Excluded for: Prior enrollment or history of ARDS (each subject analyzed once, duplicate enrollment excluded) (n = 97), non-Caucasian race (n = 99), or other reasons (n = 8).

Study criteria and exclusion criteria have been previously described [5]. Briefly, adult ICU admissions to the Massachusetts General Hospital (Boston, MA) from September 1999 to May 2005 were screened for risk factors for ARDS development including sepsis, septic shock, pneumonia, aspiration, trauma, and massive transfusion. Eligible patients were approached and enrolled prospectively after informed written consent was obtained from subjects or appropriate surrogates. Patients were excluded if they had previously been enrolled, had a history of ARDS, self- or surrogate reported a non-Caucasian race, or were on immunosuppression. After November 2000, patients on corticosteroids were no longer excluded given their increased use in sepsis. Study design is illustrated in Figure 1. Patients were screened daily until death or intensive care unit (ICU) discharge and were defined as having ARDS if they developed respiratory failure requiring intubation and met American-European Consensus Committee (AECC) criteria for ARDS [2]. A case control design was employed using ARDS patients as cases and patients without ARDS as controls. By assuming a recessive model of inheritance with a high-risk allele frequency of 0.4 and genotypic relative risk of 2, 379 patients with ARDS and 758 controls would provide 80% power. The Massachusetts General Hospital Institutional Review Board approved the study.

### Genotyping

Genomic DNA was extracted from whole blood samples using PureGene kits (Gentra Systems, Research Triangle, NC). The *NFKB1* promoter polymorphism (rs28362491) on chromosome 4q24 was genotyped using 5′ nuclease (Taqman) assays with custom primers and probes (Applied Biosystems, Foster City, CA). Genotyping was performed by laboratory personnel blinded to case-control status. All samples were successfully genotyped, and 5% of samples (59) were randomly reanalyzed for quality control. Two separate readers (RZ and LS) interpreted results, and no genotyping differences were found.

### Statistical Analysis

Hardy-Weinberg equilibrium of alleles was tested using PROC ALLELE in the SAS genetics software package (version 9.1, SAS computing). Univariate analysis was performed using chi-square tests for categorical variables and Wilcoxon Rank Sum or *t*-tests for continuous variables as appropriate. Variables were selected for inclusion into the final logistic regression model for ARDS development using a backwards stepwise selection algorithm at a threshold value of *P*>.2. Age by decade and severity of illness (APACHE III score) were forced into the model after removing PaO_2_/FiO_2_ and age from the APACHE III score to avoid co-linearity in the model [20]. Effect modification by covariates was evaluated by addition of interaction terms to the model; interaction terms were removed if they did not reach statistical significance. A recessive model was chosen for genotype (*del/del* patients vs. all other patients). We selected this model as a previous study demonstrated a statistically significant increase in ARDS severity among *del/del* patients when compared to other genotypes; a statistically significant difference was not seen when *ins/del* patients were compared to *ins/ins* patients [10].

Patients who developed ARDS were studied for secondary outcomes including 60-day mortality and organ dysfunction as determined by daily MODS score. Mortality was analyzed in a time-to-event analysis in a Cox proportional hazards model using patients with and without the *del/del* genotype as comparison groups. Age by decade and revised APACHE III score were forced into the model and the remainder of the covariates for the model were chosen using a stepwise selection algorithm as above. Covariates in the final model were tested for compliance with the proportional hazards assumption; no violations of the assumption were found. Multiple Organ Dysfunction (MODS) scores were recorded daily for 28 days or until death or ICU discharge. Average daily MODS scores over time were compared between cases and controls using repeated-measures ANOVA for unadjusted analysis, and using a mixed-effects model (PROC MIXED, SAS Software) adjusting for baseline severity of illness.

## Results

### Study Population

From October 1999 to February 2005, 18,332 adult ICU admissions were screened, and 1,376 patients were enrolled in the cohort for this study. Excluded patients included those of non-Caucasian ancestry (n = 99), those previously enrolled or with a history of ARDS (n = 97), and those excluded for other reasons such as indeterminate ancestry (n = 8). The final analysis included 379 patients with ARDS and 793 controls.

Characteristics of the study population are shown in Table 1. Patients with ARDS were significantly younger and had higher APACHE III scores than controls. They were also more likely to have septic shock, a pulmonary source of infection, to have received blood transfusion, and to have direct pulmonary injuries or multiple risk factors for ARDS. Controls were more likely to be diabetic than patients with ARDS.

**Table 1:** Characteristics of Study Population.

Characteristic	At-risk controls	ARDS	*P*-value
Total *n*	793	379	
Age (mean ± SD)	63.4±17.1	60.0±18.7	0.003
Female gender	327 (41%)	158 (42%)	0.88
APACHE III score (mean ± SD)	68.0±23.1	77.3±23.8	<0.0001
Sepsis syndrome	299 (38%)	102 (27%)	0.0003
-Pulmonary source	152 (19%)	82 (22%)	<0.0001
-Extrapulmonary source	147 (19%)	20 (5%)	
Septic shock	345 (44%)	219 (58%)	<0.0001
-Pulmonary source	165 (21%)	161 (43%)	<0.0001
-Extrapulmonary source	180 (23%)	58 (15%)	
Trauma	64 (8%)	29 (8%)	0.80
Received blood transfusion	406 (51%)	234 (62%)	0.0006
Aspiration	59 (7%)	34 (9%)	0.36
>1 risk factor for ARDS	73 (9%)	49 (13%)	0.05
Direct pulmonary injury	383 (48%)	275 (73%)	<0.0001
Diabetes	213 (27%)	67 (18%)	0.001
Alcohol abuse history	70 (9%)	49 (13%)	0.04
Serum bilirubin (mg/dL, mean ± SD)	1.2±2.9	1.8±4.0	0.008

### Genotype association with risk of developing ARDS

Genotype frequencies did not differ significantly from those predicted by Hardy-Weinberg equilibrium (p = .12) (Table 2). In all, 524 patients were heterozygous, and 176 were homozygous for the deletion allele. The calculated allele frequency was 0.39, similar to that reported in the previously published cohort [10].

**Table 2:** Genotype Frequencies.

-94ins/delATTG genotype	At-risk controls (n = 793)	ARDS (n = 379)	*P*-value
*ins/ins*	326 (41%)	146 (39%)	0.62
*ins/del*	347 (44%)	177 (47%)	
*del/del*	120 (15%)	56 (15%)	

Differences in baseline clinical characteristics by genotype are shown in Table 3. Among *del/del* homozygotes, there were no significant differences in age, sex, APACHE III score, or ARDS risk factor when compared to wild-type homozygotes and heterozygotes, but they were significantly less likely to have received blood transfusions and to be diabetic.

**Table 3:** Characteristics of Study Population by genotype.

Characteristic	*del/del patients*	All other patients	p-value
Total *n*	176	996	
Age (mean ± SD)	61.8±17.6	62.4±18.5	0.66
Female gender	67 (38%)	418 (42%)	0.33
APACHE III score (mean ± SD)	72.0±24.5	70.1±24.7	0.52
Sepsis syndrome	59 (34%)	342 (34%)	0.84
-Pulmonary source	37 (63%)	197 (58%)	0.46
-Extrapulmonary source	22 (37%)	145 (42%)	
Septic shock	84 (48%)	480 (48%)	0.91
-Pulmonary source	49 (58%)	277 (58%)	0.91
-Extrapulmonary source	35 (42%)	203 (42%)	
Trauma	9 (5%)	84 (8%)	0.13
Received blood transfusion	84 (48%)	556 (56%)	0.05
Aspiration	20 (11%)	73 (7%)	0.07
>1 risk factor for ARDS	16 (9%)	106 (11%)	0.53
Direct pulmonary injury	102 (58%)	556 (56%)	0.60
Diabetes	29 (17%)	251 (25%)	0.01
Alcohol abuse history	14 (8%)	105 (11%)	0.29
Serum bilirubin (mg/dL, mean ± SD)	1.5±3.3	1.4±2.6	0.81

Univariate analysis did not demonstrate a significant difference in ARDS risk between cases and controls according to *NFKB1* genotype (Table 2, p = 0.62). The results of the multivariate analysis of genotype association with ARDS risk are shown in Table 4. Patients homozygous for the deletion allele had greater odds of developing ARDS than did patients who were wild-type homozygous or heterozygous (adjusted odds ratio [OR_adj_] 5.21; 95% confidence interval [CI] 1.35–20.0). Covariates in the final model included age, APACHE III score, bilirubin level, receipt of any blood transfusion, presence of direct pulmonary injury, septic shock, and diabetes, as well as an interaction term between genotype and age that was found to be significantly associated with development of ARDS.

**Table 4:** Multivariate Analysis of risk of developing ARDS.

Variable	OR_adj_ *	95% CI	*p*
Genotype (*del/del*)	5.21	1.35–20.0	0.02
Age (per decade increase)	0.90	0.83–0.98	0.02
Age*genotype interaction	0.97	0.95–0.99	0.007
Apache III Score (per unit increase)	1.01	1.00–1.02	0.008
Septic Shock (yes/no)	1.80	1.35–2.41	<0.0001
Direct Pulmonary Injury (yes/no)	3.46	2.58–4.65	<0.0001
Blood transfusion (per unit received)	1.04	1.02–1.06	0.002
Diabetes history (yes/no)	0.59	0.42–0.82	0.002
Serum bilirubin (per 1 mg/dL increase)	1.03	0.99–1.08	0.12

*Odds ratio for effect on ARDS development, determined from logistic regression modeling.

The interaction between age and genotype was further investigated by stratifying the analysis according to the median age of 65. Patients under 65 had increased odds of developing ARDS in the presence of the *del/del* genotype (OR_adj_ 9.43; 95% CI 1.21–73.2, *P* = 0.03). However, this association was not observed among patients over 65 (OR_adj_ 3.60; 95% CI 0.004–>999.9, *P* = 0.71). Frequency of the *del/del* genotype was equal in both age strata (15% vs. 15%, P = 0.74). Given these results, sensitivity analysis was conducted to compare results at different stratification cut-points. Beginning with the 25^th^ percentile of age (age greater or less than 50), the analysis was repeated after advancing the stratification cut-point by one year for each iteration through the 75^th^ percentile of age (age 76). In doing so, the association between genotype and ARDS risk remained consistent in the younger age group until a cutoff of age 70, after which the association was no longer significant. In the older age group, the association remained non-significant in all iterations of the analysis.

### Genotype association with secondary outcomes

**Figure 2:**
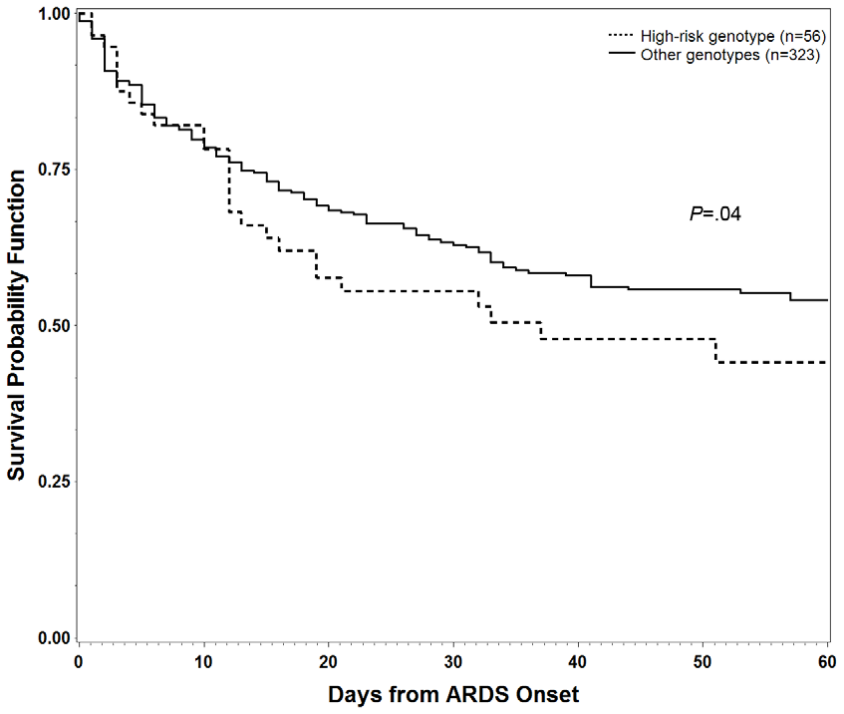
Kaplan-Meier analysis of 60-day survival by genotype. Graph depicts 60-day survival for *del/del* patients vs. all other patients. P-value stated is for Cox proportional hazards model of adjusted hazard of death within 60 days.

**Figure 3:**
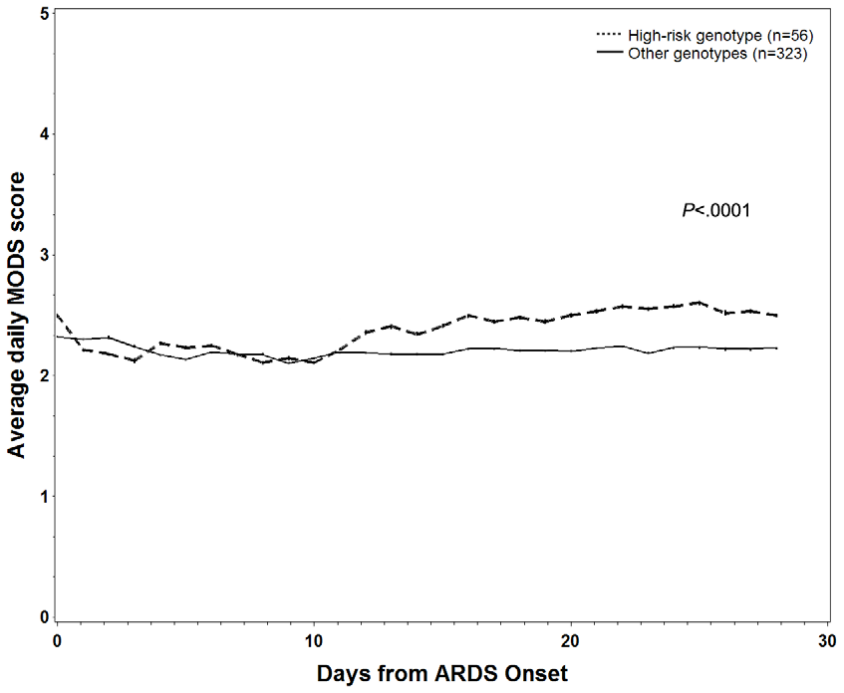
Plot of average daily MODS score by genotype. Graph depicts 28-day MODS for *del/del* patients vs. all other patients. P-value stated is for mixed effects model adjusting for APACHE III severity of illness score and preexisting organ dysfunction.

60-day mortality was higher among *del/del* patients when compared to all others (48% vs. 41%), but this was not statistically significant on univariate analysis (*P* = .22). The results of Cox proportional hazard analysis are shown in Table 5. The *del/del* genotype was associated with increased hazard of mortality at 60 days among patients with ARDS when compared to other genotypes (*P* = .04). Kaplan-Meier survival analysis is demonstrated graphically in Figure 2. When daily organ dysfunction scores were compared using repeated-measures ANOVA, *del/del* patients had significantly greater likelihood of more organ failures during their ICU stay (*P*<.0001). When this analysis was adjusted for baseline APACHE III score and presence of preexisting organ failure, the association remained significant (*P*<.0001). Daily MODS score compared by genotype are represented graphically in Figure 3.

**Table 5:** Multivariate analysis of risk of 60-day mortality (ARDS Only).

Variable	HR_adj_ *	95% CI	*p*
Genotype (*del/del*)	1.54	1.01–2.36	0.04
Age (per decade increase)	1.37	1.23–1.52	<0.0001
Apache III Score (per unit increase)	1.03	1.02–1.04	<0.0001
Blood transfusion (per unit received)	1.03	1.02–1.04	<0.0001
Trauma (yes/no)	0.09	0.012–0.64	0.02
Diabetes history (yes/no)	1.00	0.99–1.00	0.26
Aspiration (yes/no)	1.36	0.81–2.29	0.25

*Hazard ratio for 60 day mortality among ARDS patients, determined from Cox proportional hazards modeling.

## Discussion

On unadjusted analysis, our study failed to show an association between patients homozygous for the deletion allele in the promoter *of NFKB1* and an increased risk of developing ARDS. However, *del/*del patients did have an increased risk of developing ARDS in the presence of a significant interaction between genotype and age. Among patients who developed ARDS, the *del/del* genotype was associated with increased hazard of 60 day mortality and more severe daily organ dysfunction.

This study has a number of strengths. First, phenotype misclassification is minimized by prospectively determining ARDS using the AECC definition. Second, we use critically ill patients with ARDS risk factors as controls rather than healthy subjects or population-based controls. Although data collection and enrollment are simplified by use of such controls, healthy individuals, even those with a genetic predisposition to developing ARDS, would not be expected to do so in the absence of clinical risk factors. Similarly, population-based controls lack an appropriate stimulus for developing ARDS and therefore would not represent a group at equivalent risk for the disease. Including patients with different risk factors in the study reduces confounding from an association between the gene and any individual risk factor. Third, analysis was restricted to one ethnic group which minimizes the likelihood of false-positive results due to population stratification.

However, since ancestry was self-reported, false-positive results due to population stratification are still possible. Generalizations to populations other than Caucasians may also be limited. Due to the study design, the results may not be generalized to immunocompromised patients and patients with other risk factors for ARDS. Another limitation is that NF-κB activity was not measured, and the functional significance of this polymorphism is not entirely clear. Furthermore, we cannot exclude the possibility that this polymorphism is in linkage disequilibrium with the causal variant. This work represents ongoing research into the genetics of ARDS among a cohort of critically ill patients [5], [6], [8], [9], [11], [12]. Even though the association of genetic variability in *NFKB1* and risk for ARDS is biologically plausible, the risk of Type I error is increased when multiple hypotheses of genetic association are tested.

NF-κB is required for transcription of most proinflammatory molecules including cell adhesion molecules, chemokines, and cytokines. Given this central role in acute inflammatory processes, a common polymorphism in the promoter of *NFKB1* may modulate the natural course of ARDS. In an *in vitro* promoter assay, cells transfected with the deletion allele of *NFKB1* showed less activity than comparable constructs containing the insertion allele [19]. Therefore, patients with the *del/del* genotype may have decreased levels of p50.

Unlike other NF-κB members, p50 does not contain the COOH-terminal transactivation domain that is necessary for the positive regulation of gene expression. p50 may consequently form inhibitory homodimers that function as transcriptional repressors [21]. Mice lacking p50 are more sensitive to lipopolysaccharide (LPS)-induced shock as evidenced by increased TNF levels and decreased survival [22]. During bacterial pneumonia in mice, p50 deficiency increases cytokine expression and worsens lung injury [23]. Furthermore, mice lacking p50 have increased neutrophilic lung inflammation after exposure to LPS [24]. In patients with the *del/del* genotype, decreased p50 synthesis may lead to decreased repressive homodimers and increased active heterodimers of the NF-κB complex. This balance may perpetuate the inflammatory injury of ARDS.

The relationship between age, the *del/del* genotype, and the development of ARDS also requires further clarification. Age-varying genetic associations have been found and validated recently in multiple populations and are now thought to contribute to inconsistent genetic association studies for complex disease [25]. Such age related effects of genotype frequency and gene effects and age related differences in inflammatory response have been shown in multiple studies [26]–[28]. Additionally, this type of interaction has been previously described in another ARDS candidate gene study [6].

Immune dysregulation may be more likely as patients age. In patients with septic shock, TNF-α levels are higher in elderly when compared to younger individuals [29]. This propensity for exuberant inflammation may overwhelm the presumed increase in innate immunity conferred by the *del/del* genotype. In younger patients without this inflammatory diathesis, the gene effect may be more clinically relevant. However, given that the inflammatory state of our patients was not examined, this finding should be used for hypothesis generation.

In conclusion, our study demonstrates an increased risk of ARDS in patients homozygous for the deletion allele of the *NFKB1* polymorphism, with a significant interaction with age. Patients with this genotype who develop ARDS also have increased mortality and more severe organ dysfunctions. This study should be repeated in an independent population.
